# Recognition of Freezing of Gait in Parkinson’s Disease Based on Machine Vision

**DOI:** 10.3389/fnagi.2022.921081

**Published:** 2022-07-14

**Authors:** Wendan Li, Xiujun Chen, Jintao Zhang, Jianjun Lu, Chencheng Zhang, Hongmin Bai, Junchao Liang, Jiajia Wang, Hanqiang Du, Gaici Xue, Yun Ling, Kang Ren, Weishen Zou, Cheng Chen, Mengyan Li, Zhonglue Chen, Haiqiang Zou

**Affiliations:** ^1^Department of Neurosurgery, General Hospital of Southern Theater Command of PLA, Guangzhou, China; ^2^Graduate School, Guangzhou University of Chinese Medicine, Guangzhou, China; ^3^GYENNO SCIENCE Co., LTD., Shenzhen, China; ^4^Department of Neurology, The 960th Hospital of PLA, Taian, China; ^5^Department of Neurosurgery, Guangdong Second Provincial General Hospital, Guangzhou, China; ^6^Department of Neurosurgery, Ruijin Hospital, Shanghai Jiao Tong University School of Medicine, Shanghai, China; ^7^Department of Neurology, Guangzhou First People’s Hospital, Guangzhou, China; ^8^HUST-GYENNO CNS Intelligent Digital Medicine Technology Center, Wuhan, China; ^9^Branch of National Clinical Research Center for Geriatric Diseases, Chinese PLA General Hospital, Guangzhou, China

**Keywords:** Parkinson’s disease, freezing of gait, XGBoost, machine vision, machine learning

## Abstract

**Background:**

Freezing of gait (FOG) is a common clinical manifestation of Parkinson’s disease (PD), mostly occurring in the intermediate and advanced stages. FOG is likely to cause patients to fall, resulting in fractures, disabilities and even death. Currently, the pathogenesis of FOG is unclear, and FOG detection and screening methods have various defects, including subjectivity, inconvenience, and high cost. Due to limited public healthcare and transportation resources during the COVID-19 pandemic, there are greater inconveniences for PD patients who need diagnosis and treatment.

**Objective:**

A method was established to automatically recognize FOG in PD patients through videos taken by mobile phone, which is time-saving, labor-saving, and low-cost for daily use, which may overcome the above defects. In the future, PD patients can undergo FOG assessment at any time in the home rather than in the hospital.

**Methods:**

In this study, motion features were extracted from timed up and go (TUG) test and the narrow TUG (Narrow) test videos of 50 FOG-PD subjects through a machine learning method; then a motion recognition model to distinguish between walking and turning stages and a model to recognize FOG in these stages were constructed using the XGBoost algorithm. Finally, we combined these three models to form a multi-stage FOG recognition model.

**Results:**

We adopted the leave-one-subject-out (LOSO) method to evaluate model performance, and the multi-stage FOG recognition model achieved a sensitivity of 87.5% sensitivity and a specificity of 79.82%.

**Conclusion:**

A method to realize remote PD patient FOG recognition based on mobile phone video is presented in this paper. This method is convenient with high recognition accuracy and can be used to rapidly evaluate FOG in the home environment and remotely manage FOG-PD, or screen patients in large-scale communities.

## Introduction

Freezing of gait (FOG) is one of the most common motor symptoms in Parkinson’s disease (PD), mostly occurring in the intermediate and advanced stages of PD. FOG can be asymmetric, generally affecting one lower limb. Patients suddenly feel as if their feet are glued to the ground, it is difficult to lift their feet and take a step, and it is usually accompanied by a tremor in both legs (with a frequency of 6-8 Hz). This symptom usually lasts a few seconds but can sometimes exceed 30 s or last for a few minutes or more (Nutt et al., 2011). In the early stages of the disease, approximately 20% of PD patients report FOG episodes (Zhang et al., 2016), and its occurrence can increase up to 90% in the advanced stages (Hall et al., 2015). The greatest risk associated with FOG is falling. Prospective studies have revealed that 45-68% of PD patients fall each year (Wood et al., 2002; Allcock et al., 2009; Latt et al., 2009; Matinolli et al., 2011; Paul et al., 2014), and 60% of these falls are mainly caused by FOG (Pelicioni et al., 2019). Fractures from these falls can result in disability and prolonged bed rest, which can lead to a series of complications and even death. Therefore, the assessment of FOG symptoms is critical for preventive and protective measures.

Current methods for assessing FOG primarily fall into the following categories, but each method has some disadvantages:

(1) Evaluation methods based on specific gait tests or relevant rating scales (Giladi et al., 2000; Giladi et al., 2009; Nieuwboer et al., 2009; Seuthe et al., 2021): Although the rating scale evaluation method is simple and convenient, professional medical equipment and operation is not required. Therefore, this method is susceptible to environmental (Weiss et al., 2020) and subjective factors (Barthel et al., 2016). In addition, this assessment method is more difficult to implement for patients with cognitive impairments. Moreover, it is difficult to guarantee the consistency of evaluation results among different evaluators. Therefore, this method may lack high accuracy, repeatability and reliability.
(2) Evaluation methods based on neuroimaging examinations: Cerebral functional imaging technology, such as structural magnetic resonance imaging (MRI), functional MRI (fMRI), positron emission tomography (PET)/single-photon emission computed tomography (SPECT), etc., can dynamically detect cerebral functional activities and is now widely used in clinical practice (Djaldetti et al., 2018; Kim et al., 2018; Matar et al., 2019; Droby et al., 2021). These technologies provide potential imaging biomarkers for FOG research, but many researchers have only explained the correlation, not the causality, between examination results and FOG. A neuroimaging examination is considered reference data for FOG rather than an evaluation tool. In addition, the high expense and inconvenience of a neuroimaging exam make it unsuitable for clinical screening of large FOG populations.
(3) Evaluation methods based on smart devices: As objective evaluation methods, these technologies can remove the impact of human subjective factors. Currently, there are mainly two evaluation methods. The first method is based on wearable devices, and the second method is based on machine vision.

(1) Wearable device-based FOG evaluation methods: Both the sensitivity and specificity of FOG detection in PD patients based on sensor-related technology can reach a fairly high level (Sigcha et al., 2020), ideally suited for precision diagnosis in healthcare facilities. Although the current wearables are quite small in size and convenient to wear, the precision of equipment inevitably leads to an increase in cost and relatively complex installation or operation, and wearing multiple on-body sensors may induce some level of discomfort. In addition, the number, location, setting parameters and other operating points of sensors are not unified. Improper selection may lead to insufficient data collection or increase the difficulty of data analysis due to somewhat extreme environmental interference (Brognara et al., 2019), both of which pose significant challenges for wearable sensors in FOG recognition and monitoring.
(2) Machine vision-based FOG evaluation methods: Machine vision is a technology for evaluation that replaces human eyes with a machine. Compared with sensor wearables, machine vision-based methods do not require that a device be worn and neither induces discomfort in nor affects the motion of the evaluated patient; hence, it is an ideal objective evaluation method. There are two main ways to extract motion information with this method. (1) Some studies have leveraged the 3D motion capture system represented by the Kinect depth camera to extract motion information related to detecting gait disorders (Amini et al., 2019; Soltaninejad et al., 2019). However, these cameras require specialized equipment such as Microsoft Kinect, which is expensive. In addition, the complex structure of the assessment system can only be used in indoor environments, such as laboratory or clinic, resulting in a significant limitation of the evaluation environment, which is not conducive to the adoption of this technology in the screening and management of FOG populations. (2) Another method is using RGB technology of 2D keypoint recognition represented by OpenPose (Hu et al., 2019), which can estimate the joint coordinates of persons in the videos obtained using a monocular camera without external scales or markers. A study proposed a novel architecture of a graph convolutional neural network to detect FOG through 2D keypoint estimation and achieved good detection performance (Hu et al., 2020). Another study also used a deep learning method of convolutional 3D attention network to recognize FOG, which outperformed several state-of-the-art human action recognition methods (Renfei et al., 2018). The joint angles or other spatial parameters can be calculated by a home video camera or mobile phone camera at home without the special equipment needed to process 3D motion capture data. Although RGB cameras fail to cope with occluded bodies in multi-person tracking situations, RGB technology is low in cost, convenient, and easy to promote, which is very suitable for the screening of target populations in the community. Therefore, we finally chose RGB technology based on OpenPose as the preliminary scheme of our study (Viswakumar et al., 2019).

PD is a chronic disease, and patients need to occasionally go to the hospital to receive a follow-up check. However, the movements of PD patients are limited, which makes it inconvenient to visit a doctor. In addition, due to medical resource shortages, travel restrictions, and cross-infection in public places caused by the COVID-19 pandemic, it has been more difficult for PD patients to obtain diagnosis and treatment. Therefore, a remote assessment of FOG is necessary for doctors who require follow-up and management of long-term FOG patients.

To address the shortcomings associated with previous FOG recognition technologies, this study proposed constructing a FOG evaluation system based on mobile phone video. The timed up and go (TUG) test and narrow TUG (Narrow) videos of subjects were recorded by using an RGB camera, and used OpenPose to obtain the keypoint position signals of the human body and extract many representative time-domain and frequency-domain features. Then, these features were fed into the XGBoost classifier after feature selection to build the models. When modeling, we consider that a previous FOG recognition algorithm recognized FOG throughout the entire gait process, without considering the differences between the walking and turning stages. Due to the shooting angle, the visual aspects of walking and turning motions are completely different. Walking and turning gaits also vary in their kinematics. A logistic regression model determined that compared to a straight walk, turning has a reduced range of gait swing angle with significantly increased asymmetric gait and stride time (O’Day et al., 2020). Clinically, relevant brain and nervous system pathways during walking and turning are also different: turning tasks require more attention and involve greater interlimb coordination, increased coupling between posture and gait and modifications of locomotor patterns that require frontal lobe cognitive and executive functions that control posture transition (Nonnekes et al., 2019). Even under normal walking conditions, PD patients also often show longer turn times and a greater number of strides required to complete turning, which may be associated with impaired motion patterns when switching from walking to turning (Earhart, 2013). Based on the above reasons, we established motion recognition models and FOG recognition models that conform to different walking characteristics. Finally, we acquired a multi-stage FOG recognition model with acceptable performance through the leave-one-subject-out (LOSO) method, which realized automatic FOG recognition and monitoring.

## Materials and Methods

### Materials

#### Participants

The inclusion criteria were as follows: (1) participants were diagnosed with PD according to the Movement Disorder Society (MDS) diagnostic criteria (Postuma et al., 2015); (2) participants were diagnosed with FOG according to clinical manifestations and the FOG questionnaire; (3) participants were in the “on” state of medication; (4) participants could independently walk for more than 20 meters; (5) participants did not have cognitive dysfunction according to the Mini-Mental State Examination (MMSE >24 points) (Folstein et al., 1975); and (6) participants did not have any conditions affecting walking ability, such as hydrocephalus, cardiovascular and cerebrovascular disease, cognitive impairment, rheumatism, orthopedic disease, etc.

The exclusion criteria were as follows: participants with secondary PD causes, such as inflammatory, drug-induced, vascular, or toxin-induced PD, or participants with other neurodegenerative diseases, such as progressive supranuclear palsy (PSP) or multiple system atrophy (MSA) and other Parkinson-plus syndromes.

The research protocol in compliance with the Declaration of Helsinki was approved by the Scientific Ethics Committee of General Hospital of Southern Theater Command of PLA and the Ethics Committee of Guangzhou First People’s Hospital. All participants came from the General Hospital of Southern Theater Command of PLA and the Guangzhou First People’s Hospital, and written informed consent was obtained from all participants (or their legal guardians).

### Dataset

#### Video Capture

**FIGURE 1:**
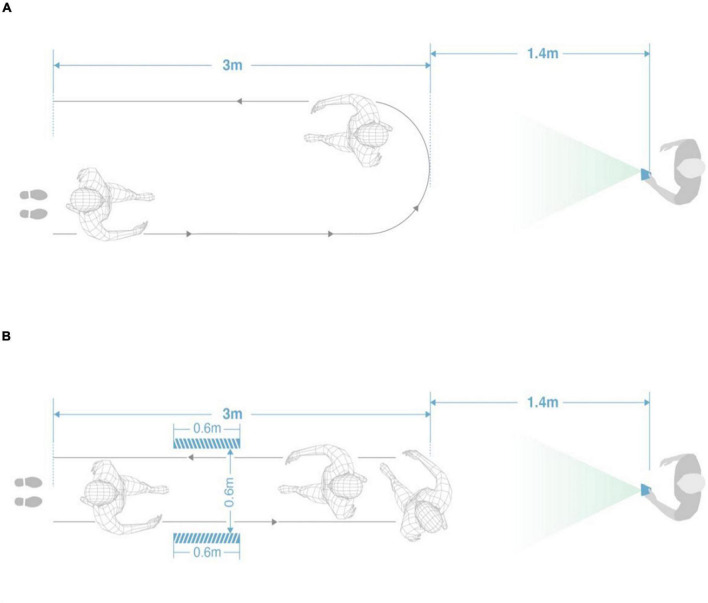
Test diagram: **(A)** TUG diagram; **(B)** narrow TUG diagram.

Each subject completed a TUG (Shumway-Cook et al., 2000) and a narrow TUG, and the test routes are shown in Figure 1. A narrow test was added because it is easier to trigger FOG in PD subjects by passing through a narrow space (Snijders et al., 2008).

The gait test process of this study was as follows: The photographer was more than 4.4 m away from the patient and recorded the videos with a mobile phone [Huawei P40 (8G + 256G)]. The frame rate of the video was 30 frames per second, with a resolution of 544*960.

After the photographer gave walking instructions, the patient started to walk, and at the same time, the photographer began to record with the mobile phone. The patient walked 3 meters in a straight line at a comfortable and free speed, turned back at the end point, and finally returned to the starting point. A narrow TUG is designed to induce FOG more easily by setting up a narrow 0.6-meter tunnel during walking. The whole process is repeated twice.

To guarantee consistency and quality of test data, the experiment was performed in an open field, which ensured that the patient was in the video during the whole recording. To prevent the subjects from falling, the participants could use a walking stick, walking aid and other auxiliary tools. The participants with a high risk of falling were accompanied by a caregiver during the walk.

#### Clinical Information Record

After subjects completed the above gait tests, we collected their clinical information, including MDS-United Parkinson’s Disease Rating Scale (UPDRS) Part 3 scores (Stebbins et al., 2013), Hoehn & Yahr ratings (stage 1-5) (Hoehn and Yahr, 1967), New Freezing of Gait Questionnaire (NFOG-Q) scores (Giladi et al., 2000) and Parkinson’s Disease Questionnaire-39 (PDQ-39) scores (Moore et al., 2007).

#### Video Annotation

To build a binary classification model, we independently annotated the turning stage and FOG stage. The video annotations of the turning and FOG stages were frame-based.

Regarding the annotation of the turning stage, the data reviewer annotated the turn-start frame and the turn-end frame. The annotation of the FOG stage was completed by two independent movement disorder specialists, and this served as the gold standard evaluation for FOG detection. More specifically, two independent neurologists separately identified the start and end of FOG episodes, and in case of discrepancies, a third neurologist was invited to make a joint decision. The three neurologists performed a common assessment to resolve any ambiguities. The start of the FOG episode was defined when the patient hesitated for more than one second at the start of walking or if it looked as if he or she was unsuccessfully trying to initiate or continue locomotion. A transient and clinically significant break in locomotion without any apparent reason was also defined as a freezing episode. The end of an episode was defined as the time when an effective step had been performed with a relatively normal length and swing, and the step also had to be followed by a continuous normal walk (Schaafsma et al., 2003). We recorded the entire gait test, such that the data were collected from the beginning of the gait test after the subjects heard the instructions to the end of the gait test. Due to the complexity of the definition of FOG, it is difficult to label all real FOG in practice. Based on the recognition of FOG for labeling purposes, we labeled the parts of the video that could be marked as FOG with certainty, and the parts of the video where it was unclear whether it was FOG would be labeled as unknown. For example, when an uninstructed stop occurred, we cannot tell whether the individual stopped to rest or FOG occurred, and these instances were marked unknown.

The dataset for this study was composed of the video of the subjects’ gait tests, the recorded scale information, and the video annotation.

### Methods

**FIGURE 2:**
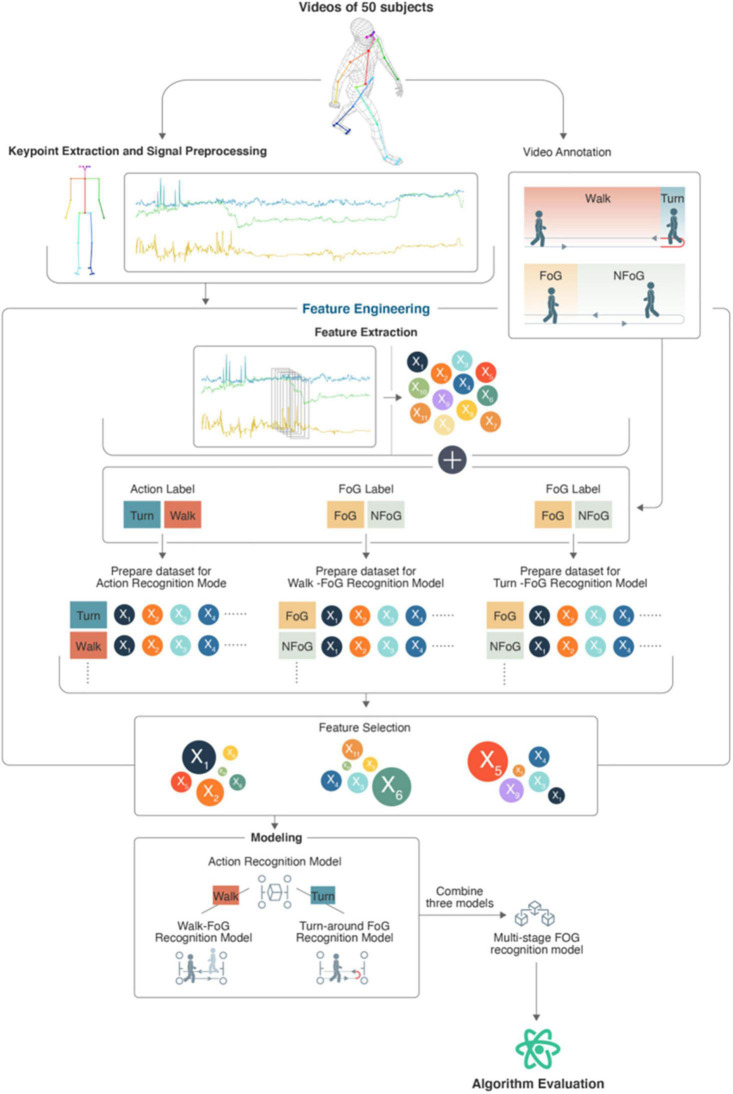
Algorithm flowchart.

The five steps of this study were keypoint position signal extraction, signal preprocessing, feature extraction, modeling and algorithm evaluation. The algorithm flowchart is shown in Figure 2.

#### Keypoint Position Signal Extraction

After videotaping the subjects’ tests *via* mobile phone, we used OpenPose (Cao et al., 2021) to extract position signals of 25 keypoints of the human skeleton to conduct 2D human motion perception. The OpenPose human gesture recognition project is an open-source library developed by US Carnegie Mellon University based on a convolutional neural network and supervised learning with Caffe as the framework that achieves human body motion, facial expression, finger movement and other posture estimates easily and accurately from images.

#### Signal Preprocessing

Human movement mainly has frequency components between 0 and 20 Hz. In addition, most previous research on FOG recognition based on wearable sensors used a 15-Hz lowpass filter for denoising (Rodriguez-Martin et al., 2017; Sigcha et al., 2020). According to the Nyquist–Shannon sampling theorem (Nyquist, 1928; Shannon, 1949), a 30-Hz sampling frequency will sample motion information below 15 Hz. A higher sampling frequency will result in more high-frequency information, but there will be additional mobile requirements that are not conducive to future applications.

**FIGURE 3:**
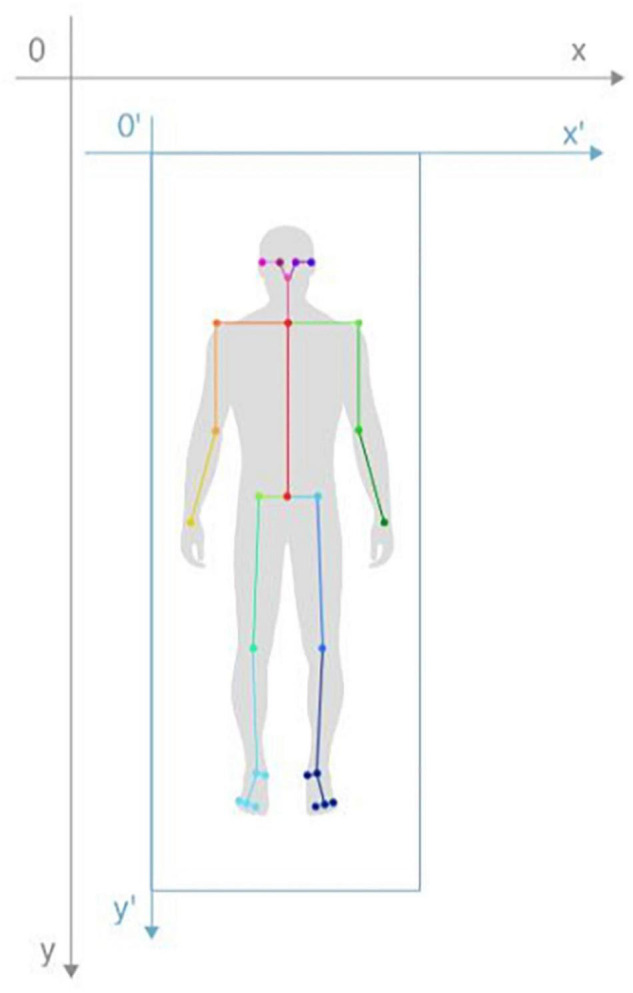
Keypoint position signal normalization diagram.

2D images have the problem of objects being near appear large and those that are far appear small, which results in different scales at different distances. Thus, we normalized the keypoint position signals from the subjects’ bodies to eliminate the effects of the different scales. First, we calculated the minimum enclosing rectangle of the human body based on 25 keypoints and expanded the length and width by 30%; then, we converted the original coordinate system to a coordinate system with the upper left corner vertex of the enclosing rectangle as the coordinate origin to obtain the position coordinates of the 25 keypoints after coordinate system conversion. Next, a ratio of 80 to the height of the enclosing rectangle was regarded as the scale factor, and this scale factor was multiplied by the position coordinate of each keypoint to obtain the normalized position coordinates. The keypoint position signal normalization diagram is shown in Figure 3.

Based on the normalized position coordinates of the keypoints in each frame, we calculated the speed signal, acceleration signal, and knee joint angle signal from the keypoints, and we calculated the absolute value signals of 8 pairs of keypoint position differences (right hip and left hip, right knee and left knee, right ankle and left ankle, right big toe and left big toe, right shoulder and left shoulder, right elbow and left elbow, right wrist and left wrist, and right ear and left ear). Among these measures, the speed signal reflects the subject’s movement speed, and the acceleration signal reflects the subject’s change in velocity. When the subject has FOG, leg movements become more obvious. By adding the angle signals from the subject’s knees, bending information of the leg joint can be extracted. Furthermore, the absolute value signals of 8 pairs of keypoint position differences showed a trend of first decreasing and then increasing when turning around, and thus, reflected the turning process.

#### Feature Extraction

Before feature extraction, we first performed sliding window processing on the signal. Sliding window signal processing is a routine processing operation, and we will not focus on this as the research priority. According to previous studies, we concluded that a window size of 2 s would yield good results (Zach et al., 2015); we adopted a sliding window with a step length of 0.1 s and a window size of 2 s to convert the signal into windows and calculated the time-domain and frequency-domain features of each window.

With respect to the motion recognition model, we extracted the minimum value, crest factor, frequency amplitude peak value, center frequency and other time-domain and frequency-domain features to reflect the differences between the walking and turning stages. With respect to the FOG recognition model, we also extracted time-domain and frequency-domain features and added features such as the freezing index (FI), the area under the power spectrum of multiple frequency bands including low-frequency bands and high-frequency bands, as well as features that may contribute to the recognition of FOG. The FI was defined as the power in the “freeze” band (3-8 Hz) divided by the power in the “locomotor” band (0.5-3 Hz). Some researchers (Moore et al., 2013) proposed that the FI can detect FOG episodes based on changes in the inertial signal power spectrum. In addition, Bächlin et al. (2009) used the power band (0.5-8 Hz) to avoid false detections when standing. In clinical observation, FOG is frequently accompanied by fast alternating trembling movements (a large increase in power in the ‘freeze’ band) of the lower extremity with both feet on the floor (Schaafsma et al., 2003; Nutt et al., 2011). The “locomotor” band is the energy band representing low frequency. It can be used to discriminate FOG (i.e., power is significantly decreased or absent in the locomotor band) from a normal gait (i.e., most of the power is in the locomotor band). Thus, FI was considered to be beneficial for FOG recognition, and this feature was added to the feature set.

#### Modeling

Next, a motion recognition model, the Walk-FOG recognition model and Turn-FOG recognition model, were built based on the collated data.

When building the motion recognition model, an excessive number of model features may lead to overfitting or other problems. As such, we carried out feature selection to improve the generalization ability of the classifiers and reduce the time required to train the classifier. First, we calculated the information gain of each feature based on XGBoost (Chen and Guestrin, 2016); the larger the information gain of the feature is, the greater the contribution of this feature to the model. Then, we used the forward feature selection strategy based on the information gain to select the best feature set. In addition, we searched for optimal model parameter combinations using grid search and the LOSO method to obtain the optimal XGBoost-based motion recognition model. The model hyperparameters for the search include the learning rate, the number of estimators (n_estimators), the max depth (max_depth), the sampling ratio (subsample), the feature sampling ratio for each tree (colsample_bytree).

**FIGURE 4:**
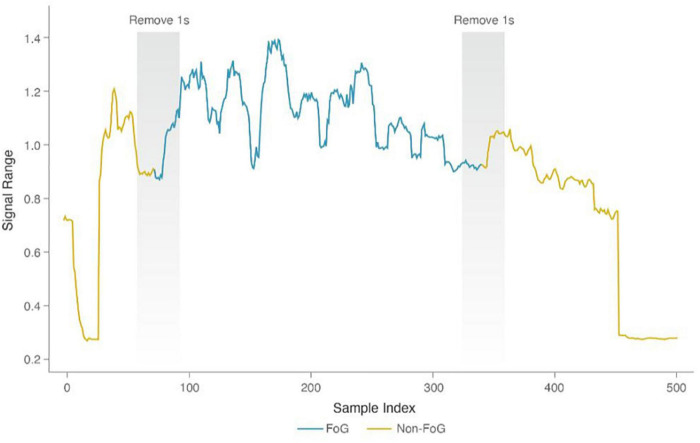
Removal of the transition samples between FOG and non-FOG.

When building FOG recognition models for the walking and turning stages, due to unbalanced samples caused by sudden FOG, we first adopted the SMOTE (Chawla et al., 2002) algorithm to balance the samples in the training set. Then, we performed the same feature selection strategy as we did with the motion recognition model. Considering that the difficulties in distinguishing samples at the transition stages are non-FOG or FOG, if they are entered during model training, the results will be affected by ambiguous labels, and performance would decline. To obtain a more precise classifier model for the FOG and non-FOG classes, we removed the samples at the transition stages of FOG and non-FOG for training but not for testing (i.e., the samples with a duration of 0.5 s for FOG and non-FOG were removed, as shown in Figure 4) (Mazilu et al., 2013). Finally, we obtained the optimal model parameter combination of the XGBoost-based FOG recognition model using grid search and the LOSO method, thereby acquiring the optimal Walk-FOG recognition model and Turn-FOG recognition model. The model hyperparameters for the search were the same as those for the motion recognition model.

#### Multi-Stage Freezing of Gait Recognition Model

We applied the motion recognition model to segment the walking and turning stages and utilized the Walk-FOG recognition model and Turn-FOG recognition model for FOG recognition in the walking and turning stages, respectively. With the strategy of first segmenting the motion stages and then recognizing FOG, we formed a multi-stage FOG recognition model. The evaluation of the FOG recognition model was episode-based. A FOG sequence composed of continuous FOG windows was considered a FOG episode; a non-FOG sequence composed of continuous non-FOG windows was considered a non-FOG episode.

Some short-lived false positive (FP) FOG events occasionally appeared in the recognition results. Therefore, with the aim of reducing false detections, we modified the recognition results of the window. Specifically, we took the 10% quantile of all the marked FOG durations as the threshold and used this value as the minimum duration necessary for a FOG episode. If the duration of a FOG episode was less than this value, we removed this FOG episode.

### Algorithm Evaluation

#### Evaluation Metrics of the Motion Recognition Model

Motion recognition model evaluation metrics are window-based sensitivity, specificity, accuracy, geometric mean (GM) and area under the curve (AUC). GM is the square root of sensitivity and specificity, and this indicator has the beneficial property of averaging out sensitivity and specificity scores while penalizing unbalanced pairs. AUC is the area under the receiver operating characteristic (ROC) curve, a common indicator used to evaluate the performance of a classification model; the higher the AUC value is, the better the model performance.

#### Evaluation Metrics of the Walk-FOG Recognition Model, Turn-FOG Recognition Model and Multi-Stage FOG Recognition Model

The evaluation metrics of these three models are episode-based sensitivity, specificity, accuracy and GM. Sensitivity is defined as the proportion of the number of correctly predicted FOG episodes (at least one window is predicted as FOG in FOG episodes) to the total number of FOG episodes; specificity is defined as the proportion of the number of correctly predicted non-FOG episodes (no window is predicted as FOG in non-FOG episodes) to the total number of non-FOG episodes; accuracy is the proportion of the number of correctly predicted episodes to the total number of episodes; GM is the square root of sensitivity and specificity.

#### Evaluation Method

Previous studies (Mazilu et al., 2012; Tripoliti et al., 2013; Sato et al., 2019), despite high sensitivity and specificity, all have some obvious problems in their evaluation methods. For example, these evaluation methods include leave-one-window-out, which trains the model using all windows except one window. Therefore, training samples with high similarity to the test samples are used for model training. With suspected data leakage, the accuracy is very high. To prevent the model evaluation results from being unrealistically high, we used the LOSO method to evaluate the performance of this algorithm on this dataset. LOSO is one of several cross-validation methods; our dataset contains 50 subjects, therefore, there is 50-fold cross-validation. For each fold of cross-validation, the data from one subject are reserved for testing, and the data of the remaining subjects are used for training. This validation method can guarantee that the data of each subject only appear in the training set or test set, which helps to statistically estimate the performance for unseen subjects (Das et al., 2011; Rodriguez-Martin et al., 2017; Hu et al., 2019).

#### Statistical Analysis

All continuous demographic data and clinical data are expressed as the mean ± SD or as a percentage. Boxplots (Toit et al., 1986) are used to explore the correlations between the walking and turning stages, FOG and non-FOG stages and features. We adopted the Wilcoxon rank-sum test to analyze whether the features used in the model analysis significantly differed between groups, such as feature differences between the walking and turning stages and differences between the FOG and non-FOG stages. Considering that the sample size was large (>30), that there were individual differences among samples, and that there were autocorrelations between time series samples, we designed a hypothesis testing method suitable for this case. First, we calculated the mean value of the feature for each subject in the two groups to obtain two sets of paired samples from the same subject; then, the paired sample Wilcoxon test was used to test whether these features of the subjects differed between groups. All figure generations and statistical analyses were performed by R 4.1.0 or Python 3.8.

## Results

### Dataset

**FIGURE 5:**
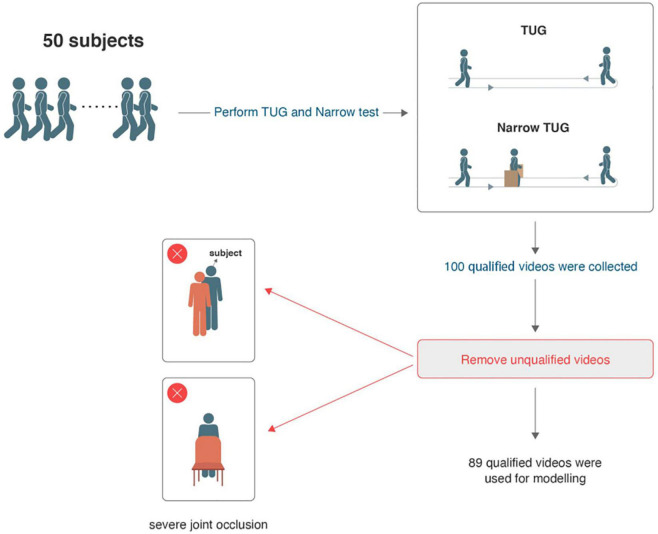
Dataset collection flowchart.

This study included convenience sample 50 PD-FOG subjects who met the criteria. Their basic information is shown in Table 1. Each subject was videotaped in the TUG and narrow TUG, resulting in a total of 200 videos being collected. After eliminating the problems of incomplete video shooting, such as video jitter or walking routes that did not conform to the requirement, 100 qualified videos were selected and further excluding unqualified videos that contained severe joint occlusion and incorrect skeleton recognition, 89 qualified videos were used for modeling (a flowchart describing the dataset collection is shown in Figure 5). The total length of the videos was 50.78 min; from this time period, the length of video including turning was 12.88 min, and that including walking was 37.9 min. Thirteen subjects had FOG during the gait test (26%), and a total of 33 FOG episodes were captured, including 25 FOG episodes in the walking stage and 8 in the turning stage, as shown in Table 2; the median FOG duration was 8.2 s (0.9-66 s).

**TABLE 1:** Demographics of the subjects.

	Values

**Number**	**50**
Age, years	71.44 ± 6.88
Sex	58% male; 42% female
Education, years	8.78 ± 4.3
Years since diagnosis	7 ± 4.61
Hoehn and Yahr stages	12.00%, stage 2.0
	48.00%, stage 2.5
	36.00%, stage 3.0
	4.00%, stage 4.0
UPDRS III (3.10 + 3.11) scores	3.22 ± 2.12
N-FOGQ scores	21.8 ± 4.39
Levodopa Equivalent Dose taken, mg/d	603.95 ± 189.23
Number of falls in past 12 months	6.6 ± 2.92

**TABLE 2:** Frequency of FOG in 13 PD patients.

FOG patient	1	2	3	4	5	6	7	8	9	10	11	12	13	Total
FOG episodes in the walking stage	2	2	2	1	2	1	3	3	3	1	1	2	2	25
FOG episodes in the turning stage	0	0	1	0	1	1	1	0	0	0	2	1	1	8
Total	2	2	3	1	3	2	4	3	3	1	3	3	3	33

### Feature Analysis

According to the forward feature selection strategy based on the information gain of XGBoost, 50, 20, and 12 features were selected for the motion recognition model, Walk-FOG recognition model, and Turn-FOG recognition model, respectively. We present these features and performed analyses.

#### Motion Recognition Model

**FIGURE 6:**
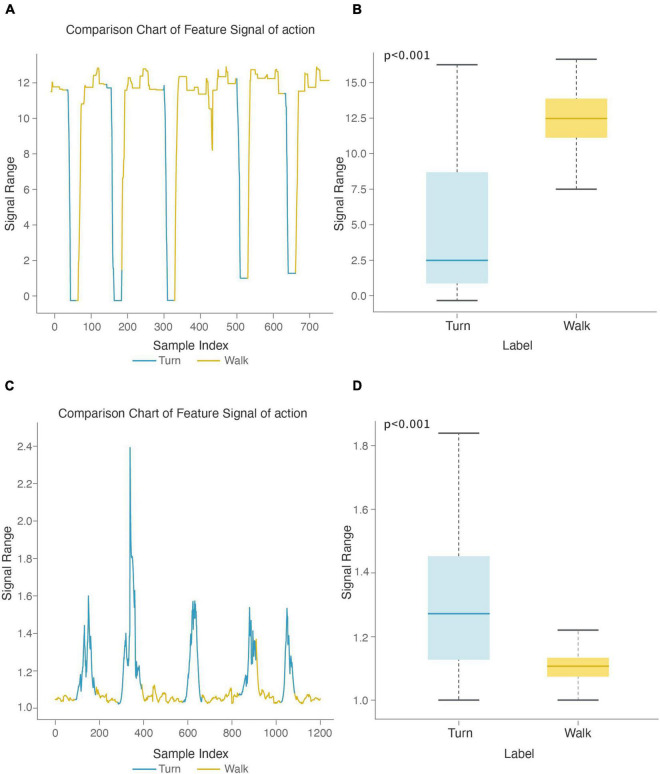
Motion feature recognition boxplot and stage comparison chart analysis. **(A)** The stage comparison chart of the minimum value calculated by the absolute value signal of the position difference between the left and right shoulders. **(B)** Boxplot of the minimum value calculated by the absolute value signal of the position difference between the left and right shoulders. The feature was significantly different between the turning and walking groups (*p* < 0.001). **(C)** Stage comparison chart of the crest factor calculated by the absolute value signal of the position difference between the right wrist and the left elbow. **(D)** Boxplot of the crest factor calculated by the absolute value signal of the position difference between the right wrist and the left elbow. The feature was significantly different between the turning and walking groups (*p* < 0.001).

After feature selection, the top 50 features of information gain were selected to build the model, of which the top 10 are shown in Table 3. Six of the top 10 features were obtained by calculating the absolute value signal of keypoint position differences, which shows that such signals best reflected the difference between walking and turning motions. To analyze the contribution of particular features to the model, we enumerated two important features used in motion recognition models and showed the stage comparison chart and boxplot, observed their numerical changes across the two stages, and analyzed the differences between the walking and turning groups. The stage comparison chart and boxplot of the minimum value (top 1) were calculated based on the absolute value signal of the position difference between the left and right shoulders in the motion recognition model (Figures 6A,B). As shown in Figure 6A, the numerical value of this feature decreased sharply when turning because the absolute value signal of the keypoint position difference first decreases to 0 and then increases during the turning process. As a result, the minimum value of this signal also showed the same change; as shown in Figure 6B, the overall numerical value of this feature when walking was significantly greater than that when turning, with a statistically significant difference (*p* < 0.001). Additionally, the numerical range while walking was smaller than that while turning, indicating that the feature fluctuated less when walking. The stage comparison chart and boxplot of the crest factor (top 6) were calculated by the absolute value signal of the position difference between the right elbow and the left elbow in the motion recognition model (Figures 6C,D). The crest factor is the ratio of the maximum value to the root mean square. Figure 6C shows that the numerical value of this feature increased when turning because the maximum value of this signal was greater than the degree of fluctuation in the signal. Figure 6D shows that the overall numerical value of this feature when turning was apparently greater than that when walking, with a significant difference between groups (*p* < 0.001). In conclusion, we believe that these features show synchronous changes and significant numerical differences when walking and turning, which markedly contributed to motion classification of the model and could facilitate subsequent research.

**TABLE 3:** Feature importance for the motion recognition model based on the XGBoost gain (top 10).

Signal	Feature	Equation	Gain
Difference in position signal between L and R shoulder (x-Axis)	Min	min(*x*)	5031.12
Difference in position signal between L and R ear (x-Axis)	90th percentile	*x*_*1+0.9(n–1)*_	691.14
Acceleration of R big toe (x-Axis)	Min MI	$min\left(x_{i}^{2}\right)$	594.00
Speed signal of L ear (x-Axis)	Skewness	$E\left[{\left(\frac{x_{i}-\mu }{\sigma }\right)}^{3}\right]$	570.47
Position signal of R big toe (x-Axis)	Mean of absolute values	$\frac{\sum_{i=1}^{n}\left|x_{i}\right|}{n}$	558.83
Difference in position signal between L and R elbow (x-Axis)	Crest factor	$\frac{max\left(x\right)}{\sqrt{\frac{\sum_{i=1}^{n}x_{i}^{2}}{n}}}$	458.90
Difference in position signal between L and R elbow (x-Axis)	Frequency amplitude peak	*max*(|*F*.*T*.(*x*)|)	448.97
Difference in position signal between L and R hip (x-Axis)	Frequency amplitude peak	*max*(|*F*.*T*.(*x*)|)	434.09
Difference in position signal between L and R wrist (x-Axis)	Frequency amplitude peak	*max*(|*F*.*T*.(*x*)|)	406.28
Speed signal of L ear (x-Axis)	Mean	$\frac{\sum_{i=1}^{n}x_{i}}{n}$	397.45

*F.T. (x) is the continuous Fourier transform of x. MI is Mutual Information.*

#### Freezing of Gait Recognition Model

Similarly, we selected the top 20 features according to the information gain of the XGBoost algorithm to build the Walk-FOG recognition model (Table 4), and the top 12 features were used to build the Turn-FOG recognition model (Table 5). Comparing the features of the Walk-FOG recognition model and Turn-FOG recognition model, we found a great discrepancy between the important features, which illustrates the necessity of staged recognition for FOG from the perspective of selected features. We found that the features of the left and right limbs were different, which may be because the results were data-driven, and the samples were not balanced on FOG of the left and right limbs.

**TABLE 4:** Feature importance for the Walk-FOG recognition model based on the XGBoost gain (top 20).

Signal	Feature	Equation	Gain
Acceleration of R heel (*y*-Axis)	0.5-3 Hz band area	$\int_{0.5}^{3}|F.T.\left(x\right){|}^{2}dx$	10789.28
Position signal of L wrist (*x*-Axis)	Average frequency	$\frac{\sum_{i=1}^{n}F.T.\left(x\right)}{n}$	1680.95
Position signal of L wrist (*x*-Axis)	Max	max(*x*)	1541.93
Difference in position signal between L and R wrist (*x*-Axis)	Clearance factor	$\frac{max\left(x\right)}{\sqrt{\frac{\sum_{i=1}^{n}x_{i}^{2}}{n}}}$	1143.63
Difference in position signal between L and R wrist (*x*-Axis)	90th percentile	*x*_*1+0.9(n–1)*_	1120.62
Speed signal of L elbow (*y*-Axis)	Sample entropy	$-\sum_{i=1}^{n}p\left(x_{i}\right)logp\left(x\right)$	953.16
Difference in position signal between L and R ankle (*x*-Axis)	Average of absolute value	$\frac{\sum_{i=1}^{n}\left|x_{i}\right|}{n}$	944.84
Speed signal of L heel (*y*-Axis)	1-1.5 Hz band area	$\int_{1}^{1.5}|F.T.\left(x\right){|}^{2}dx$	907.36
Difference in position signal between L and R knee (*x*-Axis)	Average frequency	$\frac{\sum_{i=1}^{n}F.T.\left(x\right)}{n}$	901.64
Position signal of L ankle (*y*-Axis)	1-1.5 Hz band area	$\int_{1}^{1.5}|F.T.\left(x\right){|}^{2}dx$	897.85
Position signal of L heel (*y*-Axis)	1-1.5 Hz band area	$\int_{1}^{1.5}|F.T.\left(x\right){|}^{2}dx$	884.34
Difference in position signal between L and R ankle (*x*-Axis)	Second peak of power spectral	$2ndmax\left(\int_{-\infty }^{\infty }|F.T.\left(x\right){|}^{2}dx\right)$	874.42
Acceleration signal of L elbow (*x*-Axis)	Max	max(*x*)	863.18
Position signal of L small toe (*y*-Axis)	Max	max(*x*)	855.24
Position signal of L ankle (*y*-Axis)	Min	min(*x*)	835.73
Position signal of L big toe (*y*-Axis)	Max	max(*x*)	897.85
Speed signal of L elbow (*x*-Axis)	Second peak of power spectral	$2ndmax\left(\int_{-\infty }^{\infty }|F.T.\left(x\right){|}^{2}dx\right)$	790.58
Difference in position signal between L and R ankle (*x*-Axis)	Clearance factor	$\frac{max\left(x\right)}{\sqrt{\frac{\sum_{i=1}^{n}x_{i}^{2}}{n}}}$	746.21
Speed signal of L elbow (*x*-Axis)	Min	min(*x*)	721.21
Difference in position signal between L and R ankle (*x*-Axis)	MSE	$\sqrt{\frac{\sum_{i=1}^{n}x_{i}^{2}}{n}}$	706.16

*F.T. (x) is the continuous Fourier transform of x.*

**TABLE 5:** Feature importance for the turn-FOG recognition model based on the XGBoost gain (top 12).

Signal	Feature	Equation	Gain
Speed signal of R ankle (*x*-Axis)	0.5-3 Hz band area	$\int_{0.5}^{3}|F.T.\left(x\right){|}^{2}dx$	3839.94
Acceleration of R heel (*x*-Axis)	0.5-3 Hz band area	$\int_{0.5}^{3}|F.T.\left(x\right){|}^{2}dx$	3647.17
Speed signal of L heel (*x*-Axis)	0.5-3 Hz band area	$\int_{0.5}^{3}|F.T.\left(x\right){|}^{2}dx$	3593.09
Speed of L ankle (*y*-Axis)	0.5-3 Hz band area	$\int_{0.5}^{3}|F.T.\left(x\right){|}^{2}dx$	2778.23
Position signal of R small toe (*y*-Axis)	Min	min(*x*)	1134.35
Difference in position signal between L and R elbow (*x*-Axis)	Clearance factor	$\frac{max\left(x\right)}{\sqrt{\frac{\sum_{i=1}^{n}x_{i}^{2}}{n}}}$	758.84 6
Difference in position signal between L and R ear (*x*-Axis)	Kurtosis coefficient	$\frac{\sum_{i=1}^{n}x_{i}^{4}}{n}$	7.43
Position signal of L shoulder (*x*-Axis)	Kurtosis coefficient	$\frac{\sum_{i=1}^{n}x_{i}^{4}}{n}$	619.95
Position signal of neck (*y*-Axis)	Kurtosis coefficient	$\frac{\sum_{i=1}^{n}x_{i}^{4}}{n}$	508.16
Difference in position signal between L and R wrist (*x*-Axis)	3.5-15 Hz band area	$\int_{3.5}^{15}|F.T.\left(x\right){|}^{2}dx$	445.99
Position signal of R shoulder (*y*-Axis)	Kurtosis coefficient	$\frac{\sum_{i=1}^{n}x_{i}^{4}}{n}$	426.95
Difference in position signal between L and R hip (*x*-Axis)	Peak-to-peak value	*max*(*x*)−*min*(*x*)	423.31

*F.T. (x) is the continuous Fourier transform of x.*

As shown in Tables 4, 5, many features used for building the Walk-FOG recognition model and Turn-FOG recognition model were the area under the power spectrum of the 0.5-3 Hz frequency band calculated from various signals, which are conducive to FOG recognition. The 0.5-3 Hz frequency band is the energy band representing low frequency. As Moore et al. (2013) showed in their study, the area under the power spectrum in this band increased during normal walking and decreased during FOG. They also showed a decrease in FI when patients experienced FOG, which is consistent with the reduced area under the power spectrum of the 0.5-3 Hz frequency band as the denominator in the calculation of FI. The “locomotor” band (0.5-3 Hz) of the FI is useful for recognizing FOG. In addition, the Turn-FOG recognition model used the features that included the area under the power spectrum of the 3.5-15 Hz frequency band (Table 5), and this result was similar to that of Moore et al. (2013). They found that the area under the power spectrum of the 3-8 Hz high frequency band increased when subjects experienced FOG.

**FIGURE 7:**
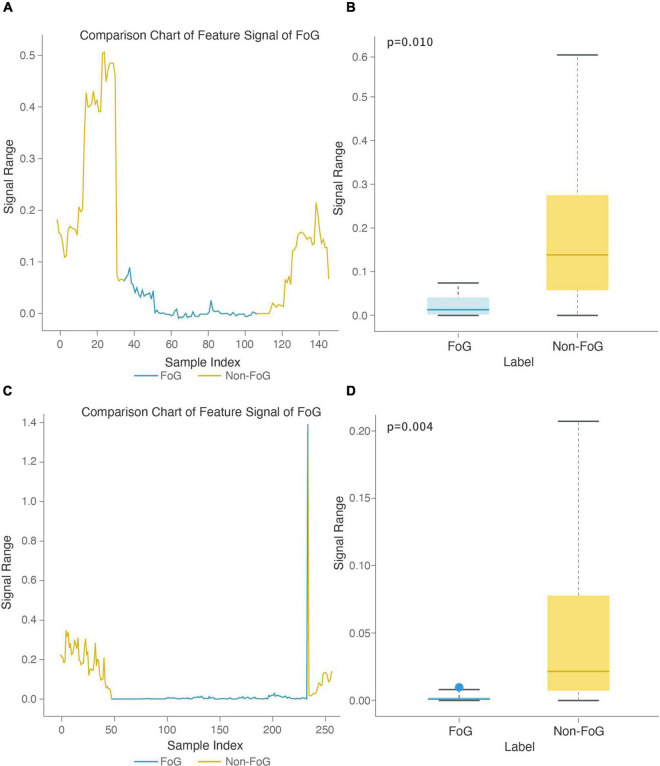
Feature boxplot and stage comparison chart analysis in the walking stage. **(A)** Stage comparison chart of the area under the power spectrum of the 0.5-3 Hz frequency band calculated by the *Y*-axis acceleration signal from the right heel. **(B)** Boxplot of the area under the power spectrum of the 0.5-3 Hz frequency band calculated by the *Y*-axis acceleration signal from the right heel. The feature was significantly different between the FOG and non-FOG groups (*p* = 0.010). **(C)** Stage comparison chart of the area under the power spectrum of the 1-1.5 Hz frequency band calculated by the *Y*-axis speed signal from the left heel. **(D)** Boxplot of the crest factor calculated by the area under the power spectrum of the 1-1.5 Hz frequency band calculated by the *Y*-axis speed signal from the left heel. The feature was significantly different between the FOG and non-FOG groups (*p* = 0.004).

To further illustrate the contribution of features to the FOG recognition model, we analyzed two features used in the Walk-FOG recognition model and Turn-FOG recognition model and presented them in a visualized manner. The stage comparison chart and boxplot of the area under the power spectrum of the 0.5-3 Hz frequency band (top 1) was calculated based on the *Y*-axis acceleration signal from the right heel in the Walk-FOG recognition model (Figures 7A,B). The 0.5-3 Hz frequency band represents the low-frequency energy band. When FOG occurs, there is no significant movement of the subject’s legs, so the low-frequency energy will be considerably reduced. Therefore, as shown in Figure 7A, the numerical value of this feature was significantly reduced and remained low for a long time; as shown in Figure 7B, the numerical value of this feature was very low when FOG occurred, and there was a significant numerical difference between the FOG and non-FOG groups (*p* = 0.010). The stage comparison chart and boxplot of the area under the power spectrum of the 1-1.5 Hz frequency band (top 8) was calculated based on the *Y*-axis speed signal from the left heel in the Walk-FOG recognition model (Figures 7C,D). The 1-1.5 Hz frequency band is also a low-frequency energy band. When the subject has FOG, the energy in this frequency band also decreased. As shown in Figure 7C, this feature was reduced to close to zero when FOG occurred. Moreover, there was also a significant numerical difference between the FOG and non-FOG groups (*p* = 0.004) (Figure 7D).

**FIGURE 8:**
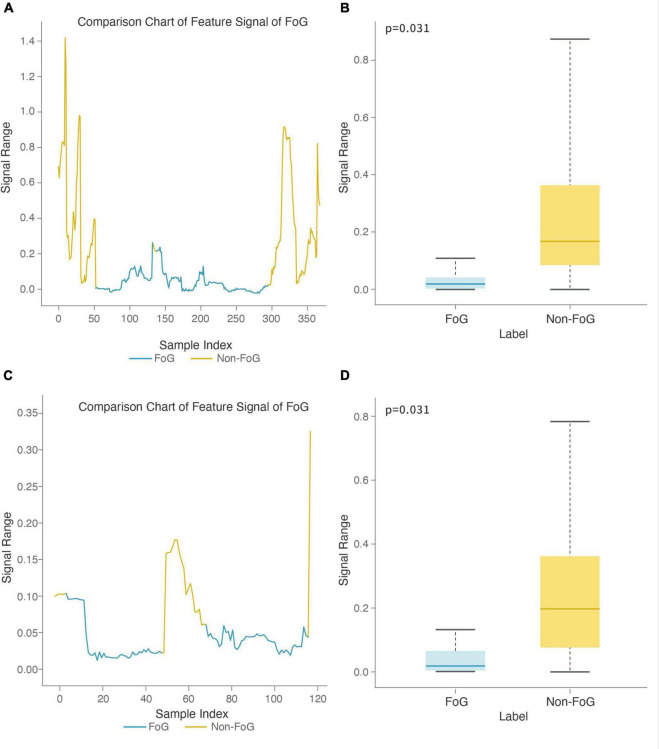
Feature boxplot and stage comparison chart analysis in the turning stage. **(A)** Stage comparison chart of the area under the power spectrum of the 0.5-3 Hz frequency band calculated by the *X*-axis acceleration signal from the right heel. **(B)** Boxplot of the area under the power spectrum of the 0.5-3 Hz frequency band calculated by the *X*-axis acceleration signal from the right heel. The feature was significantly different between the FOG and non-FOG groups (*p* = 0.031). **(C)** Stage comparison chart of the area under the power spectrum of the 0.5-3 Hz frequency band calculated by the *X*-axis speed signal form the left heel. **(D)** Boxplot of the area under the power spectrum of the 0.5-3 Hz frequency band calculated by the *X*-axis speed signal from the left heel. The feature was significantly different between the FOG and non-FOG groups (*p* = 0.031).

The stage comparison chart and boxplot of the area under the power spectrum of the 0.5-3 Hz frequency band (top 1) was calculated based on the *X*-axis acceleration signal from the right heel in the Turn-FOG recognition model (Figures 8A,B). The stage comparison chart and boxplot of the area under the power spectrum of the 0.5-3 Hz frequency band (top 3) was calculated based on the X-axis speed signal from the left heel in the Turn-FOG recognition model (Figures 8C,D). Both features involve the area under the power spectrum of the 0.5-3 Hz frequency band. When the numerical value of this feature suddenly decreased, it indicated that the subject may have FOG. As shown in Figures 8A,C, when FOG occurred, the numerical value of this feature suddenly decreased. As shown in Figures 8B,D, both numerical values of the two features were lower when FOG occurred, with smaller overall fluctuations as well, and the P values also passed the significance test (*p* = 0.031 and *p* = 0.031, respectively).

#### Model Performance

According to the above modeling methods, the motion recognition model has a recognition sensitivity of 81.21%, specificity of 90.16% and accuracy up to 87.75% (see Table 6). We also built FOG recognition models for the walking and turning stages. The sensitivity of the Walk-FOG recognition model was 71.43%, and the specificity was 85.71%; the sensitivity of the Turn-FOG recognition model was 61.54%, and the specificity was 92.59%. Finally, we combined the motion recognition model, Walk-FOG recognition model and Turn-FOG recognition model into a multi-stage FOG recognition model, with a specific strategy of using the motion recognition model to segment different motion stages and then to use the FOG recognition model in each stage to recognize FOG. Finally, we modified the window recognition results of the FOG recognition model and obtained an episode-based sensitivity and specificity of 87.50 and 79.82%, respectively, as shown in Table 6.

**TABLE 6:** Performance of each model.

	GM	Accuracy	Sensitivity	Specificity	AUC
Motion recognition model	85.57%	87.75%	81.21%	90.16%	92.01%
FOG recognition model	80.16%	78.01%	84.38%	76.15%	–
Multi-stage recognition model	83.57%	81.56%	87.50%	79.82%	–

#### Model Comparison

We built the non-staged FOG recognition model using the same method as the staged FOG recognition model (i.e., the multi-stage FOG recognition model) and compared the performance of the two models (see Table 6). The results suggested that both the sensitivity and specificity of the staged FOG recognition model was higher by 3%, proving better recognition performance of the staged FOG recognition model than the non-staged FOG recognition model.

## Discussion

This study proposes a new method of automatically recognizing FOG based on mobile phone video. We collected 89 qualified videos from 50 PD subjects, built a multi-stage FOG recognition model, evaluated the recognition performance of this model using the LOSO method, and obtained a relatively sound result (sensitivity: 87.50%; specificity: 79.82%). Although the specificity is slightly lower than the sensitivity, community-based screening emphasizes higher sensitivity. Thus, this model meets the preliminary criteria for clinical needs (Leeflang et al., 2013; Barry et al., 2014).

The TUG, a simple and effective way to assess an individual’s basic functional mobility, includes stages where FOG is most likely to occur, such as starting, turning, and reaching goals (Shumway-Cook et al., 2000). When designing the test, we also added a 3-m narrow test to induce FOG. When considering how long to make the TUG for use in a gait analysis based on machine vision, we read the relevant literature and found that gait parameters [including step length (SL), step width (SW), step duration (SD), single-stance duration (SSD) and double-stance duration (DSD)] extracted from 3-m TUG videos can be used as an effective and stable gait quantification method (Aberg et al., 2021). A previous study segmented patients’ starting, turning, and walking tasks from the video of the 3-m TUG to classify patients based on disease status (Tang et al., 2022). In addition, a similar study formulated vision-based FOG detection from 3-m TUG videos from 45 PD patients(Hu et al., 2019). This study showed that the 3-m TUG is widely used for machine vision gait analysis, which makes the result of the study more comparative. Therefore, using a 3-m TUG is a sound choice.

Bächlin et al. (2010) and Moore et al. (2013) found that when subjects experienced FOG, the area under the power spectrum of the 0.5-3 Hz frequency band decreased, and the area under the power spectrum of the 3-8 Hz frequency band increased. Therefore, these two features were used in FOG recognition. The energy of the low frequency band reflects normal activity. When the energy of the low frequency band is reduced, the normal activity decreases, while the FOG is usually accompanied by high-frequency tremor. As a result, the high frequency band reflects the FOG. Therefore, low-frequency and high-frequency features are useful for FOG recognition. As shown in Table 5, we used the low-frequency feature, the area under the power spectrum of the 0.5-3 Hz frequency band and the high-frequency feature, the area under the power spectrum of the 3.5-15 Hz frequency band, to build the FOG recognition model. We used the XGBoost algorithm combined with the forward feature selection strategy to select the features. Many useful frequency domain and time domain features can be obtained through this method, as shown in Tables 4, 5. All the low-frequency and high-frequency features used for FOG recognition are shown in Table 8. In addition to discovering low-frequency features and high-frequency that can be used for FOG recognition as previous, we also applied time domain features (Rodriguez-Martin et al., 2017; Sigcha et al., 2020) in FOG recognition and found that some time domain features have a great contribution in the FOG recognition model, such as the clearance factor calculated by position signal of L big toe for the Walk-FOG recognition model (see Table 4) and the min calculated by the position signal of R small toe for the Turn-FOG recognition model (see Table 5). These features, which are important for detecting FOG, were data-driven.

**TABLE 7:** NFOG-Q prediction results.

Subitems	ACC ± 0	ACC ± 1
NFOGQ-3	72.00%	96.00%
NFOGQ-6	88.64%	100.00%
NFOGQ-7	75.00%	97.73%

**TABLE 8:** The low-frequency and high-frequency features for the Walk-FOG recognition model and Turn-FOG recognition model.

	Signal	Feature	Equation
Low Frequency	Acceleration of R heel (*y*-Axis)	0.5-3 Hz band area	$\int_{0.5}^{3}|F.T.\left(x\right){|}^{2}dx$
	Speed signal of L heel (*y*-Axis)	1-1.5 Hz band area	$\int_{1}^{1.5}|F.T.\left(x\right){|}^{2}dx$
	Position signal of L ankle (*y*-Axis)	1-1.5 Hz band area	$\int_{1}^{1.5}|F.T.\left(x\right){|}^{2}dx$
	Position signal of L heel (*y*-Axis)	1-1.5 Hz band area	$\int_{1}^{1.5}|F.T.\left(x\right){|}^{2}dx$
	Speed signal of R ankle (*x*-Axis)	0.5-3 Hz band area	$\int_{0.5}^{3}|F.T.\left(x\right){|}^{2}dx$
	Acceleration of R heel (*x*-Axis)	0.5-3 Hz band area	$\int_{0.5}^{3}|F.T.\left(x\right){|}^{2}dx$
	Speed signal of L heel (*x*-Axis)	0.5-3 Hz band area	$\int_{0.5}^{3}|F.T.\left(x\right){|}^{2}dx$
	Speed of L ankle (*y*-Axis)	0.5-3 Hz band area	$\int_{0.5}^{3}|F.T.\left(x\right){|}^{2}dx$
High Frequency	Difference in position signal between L and R wrist (*x*-Axis)	3.5-15 Hz band area	$\int_{3.5}^{15}|F.T.\left(x\right){|}^{2}dx$

*F.T. (x) is the continuous Fourier transform of x.*

This study realized FOG recognition algorithm with relatively high sensitivity and specificity, which has preliminary clinical feasibility. Considering that the clinical need to assess the severity of FOG, we also attempted to predict scores of the third item (FOG frequency when turning), the sixth item (longest duration of FOG appearing at the start of the step) and the seventh item (evaluation of FOG impact on walking in daily life) on the NFOG-Q that has strong clinical objectivity. In practice, after using OpenPose to extract the position signals of the subject’s keypoints based on their skeleton, we calculated time-domain and frequency-domain features. Then, we built an ordered logistic regression model and evaluated the prediction performance of the model with the LOSO method. The prediction accuracy of all three items exceeded 70% (see Table 7). For these measures, ACC ± 0 represents the accuracy for a correctly predicted score, and ACC ± 1 represents the accuracy for a prediction error of one point. To the best of our knowledge, we propose the method for NFOG-Q score prediction in our study by machine vision to evaluate the subject’s FOG severity, which has great convenience and solves the problem of bias in evaluations caused by the subjectivity associated with self-evaluations.

The limitations of this paper are as follows:

(1) Since the included subjects did not experience FOG often during shooting, there were few FOG samples. The reasons are for this are mainly due to the characteristic of the sudden occurrence of FOG, filming environment (FOG is less likely to occur in a wide hospital corridor than in a home environment) (Snijders et al., 2008), test requirements in this study (for the sake of safety and feasibility of the study, the included PD-FOG subjects were in the “ON” state of medication during the test and were less prone to FOG), and the exclusion of more severe advanced PD subjects (i.e., those no longer in the “ON” state of medication or no longer able to stand and walk). Because the patients performed the test in the “ON” state, the “ON” state FOG has not been validated to determine whether it can be generalizable to the “OFF” state. A study found that FOG in the “OFF” state can persist in the full “ON” state after sufficient dopaminergic treatment, and the “ON” state FOG may be the same as the “OFF” state FOG but requires further verification (Lucas et al., 2019).
(2) Because the videos were shot in a real scene, some factors may have affected FOG recognition. For example, the patients had irregular motions during the test (e.g., a patient stood still for a short while due to a desire to rest). To optimize this problem, some gait parameters can be considered features to identify FOG, avoiding the influence of irregular motions, such as gait speed, cadence, and time to stop or freeze. In addition, factors such as shooting angle may have affected the effectiveness of the FOG recognition model. In our study, we asked subjects to walk toward the photographer, which eliminated the problem of the shooting angle. An affine transformation will be performed to adjust to various cameras in applications.
(3) Due to the perspective nature, the increase in the shooting distance leads to a decrease in the signal-to-noise ratio (SNR) of the keypoint position signals extracted by OpenPose. However, this relationship is acceptable. Since our purpose was to verify the feasibility of machine vision methods for FOG monitoring and assessment, as long as the results showed that the model had achieved relatively good performance and met clinical expectations, optimization of the SNR was not the focus of this study. Theoretically, if the SNR can be improved, better performance can be achieved. Therefore, the SNR can be optimized in the next step.
(4) Regarding the recognition of severe patients who needed support during the test, since OpenPose can recognize multiple skeletons, if the shooting condition was ignored in actual application, the skeleton of both the patient and the caregiver would be exposed together in the video, which would interfere with FOG recognition. In response to this problem, we will study other algorithms to assist the subjects’ skeleton recognition, thus improving information extraction effectiveness.

To further increase the representativeness and higher accuracy of our FOG recognition system, future studies should evaluate a larger sample of patients (e.g., covering residential communities and inpatient wards as well as in “OFF” state FOG). In addition, we did not examine the possible impact of fewer FOG phenotypes such as trembling, akinetic, or shuffling with the algorithms (Mazzetta et al., 2019). PD-FOG patients with many different phenotypes should be included in future studies to improve the practicality of this system. We can also combine wearable sensors to accurately detect suspected PD-FOG patients to improve the accuracy of FOG recognition.

As a new technology, FOG recognition based on machine vision has many challenges. For example, levodopa (L-dopa) and other drugs can improve space- and time-related gait parameters (such as step length and speed) (Smulders et al., 2016), thus causing a large difference between home evaluation and outpatient evaluation. In this regard, we will need to address differences in the assessment of patients under the influence of drugs to make objective assessments. If this problem can be solved, FOG recognition based on machine vision will play a key role in the identification of high-risk individuals and observation of curative effects.

## Conclusion

Freezing of gait is one of the major motor symptoms of PD patients that can cause falls. Since PD patients are mostly elderly, falling results in fractures and even death. Thus, it is critical to identify methods to detect FOG and prevent patients from falling. In this study, a multi-stage FOG recognition model and the NFOG-Q score prediction model with good performance were developed. This brand-new diagnostic evaluation service not only solves the impact of individual subjectivity but also reduces the economic burden on PD patients. The current outbreak of the COVID-19 pandemic affects all aspects of healthcare. PD-FOG patients are a particularly vulnerable group who have been directly and indirectly affected by this pandemic, resulting in a lack of timely diagnosis and treatment. Fortunately, the implementation of the FOG evaluation system based on machine vision can overcome the above problems. In this way, full-process management and timely intervention in the PD-FOG population can be truly achieved.

## Data Availability Statement

The original contributions presented in this study are included in the article/supplementary material, further inquiries can be directed to the corresponding author/s.

## Ethics Statement

The studies involving human participants were reviewed and approved by the Scientific Ethics Committee of General Hospital of Southern Theater Command of PLA and the Ethics Committee of Guangzhou First People’s Hospital. The patients/participants provided their written informed consent to participate in this study.

## Author Contributions

ML and HZ provided the sample. WL, JLu, HB, JLi, JW, HD, and GX collected data from the sample. WL, XC, JZ, and HZ drafted the manuscript. XC, WZ, CC, and ZC performed the statistical analyses. JZ, YL, KR, CZ, ML, ZC, and HZ designed and supervised the study and double checked the statistical analyses. All authors contributed to the conceptual design, collection, writing, editing, and generation of figures and tables for this manuscript and approved the submitted version.

## Conflict of Interest

XC, YL, KR, WZ, CC, and ZC were employed by GYENNO SCIENCE Co., LTD. The remaining authors declare that the research was conducted in the absence of any commercial or financial relationships that could be construed as a potential conflict of interest.

## Publisher’s Note

All claims expressed in this article are solely those of the authors and do not necessarily represent those of their affiliated organizations, or those of the publisher, the editors and the reviewers. Any product that may be evaluated in this article, or claim that may be made by its manufacturer, is not guaranteed or endorsed by the publisher.

## Abbreviations

PD: Parkinson’s disease
LOSO: leave one subject out
FOG: freezing of gait
TUG: timed up and go test
Narrow: narrow gap timed up and go test
Walk-FOG recognition model: FOG recognition model for the walking stage
Turn-FOG recognition model: FOG recognition model for the turning stage.
